# CVID Associated with Systemic Amyloidosis

**DOI:** 10.1155/2015/879179

**Published:** 2015-08-04

**Authors:** Saliha Esenboga, Deniz Çagdas Ayvaz, Arzu Saglam Ayhan, Banu Peynircioglu, Ozden Sanal, Ilhan Tezcan

**Affiliations:** ^1^Division of Immunology, Department of Pediatrics, Hacettepe University Faculty of Medicine, 06100 Ankara, Turkey; ^2^Department of Pathology, Hacettepe University Faculty of Medicine, 06100 Ankara, Turkey; ^3^Department of Medical Biology and Genetics, Hacettepe University Faculty of Medicine, 06100 Ankara, Turkey

## Abstract

Common variable immunodeficiency (CVID) is a frequent primary immune deficiency (PID), which consists of a heterogeneous group of disorders and can present with recurrent infections, chronic diarrhea, autoimmunity, chronic pulmonary and gastrointestinal diseases, and malignancy. Secondary amyloidosis is an uncommon complication of CVID. We report an unusual case of a 27-year-old male patient who presented with recurrent sinopulmonary infections, chronic diarrhea, and hypogammaglobulinemia and was diagnosed with CVID. The patient was treated with intravenous immunoglobulin (IVIg) therapy once every 21 days and daily trimethoprim-sulfamethoxazole for prophylaxis. Two years after initial diagnosis, the patient was found to have progressive decline in IgG levels (as low as 200–300 mg/dL) despite regular Ig infusions. The laboratory tests revealed massive proteinuria and his kidney biopsy showed accumulation of AA type amyloid. We believe that the delay in the diagnosis of CVID and initiation of Ig replacement therapy caused chronic inflammation due to recurrent infections in our patient and this led to an uncommon and life-threatening complication, amyloidosis. Patients with CVID require regular follow-up for the control of infections and assessment of adequacy of Ig replacement therapy. Amyloidosis should be kept in the differential diagnosis when managing patients with CVID.

## 1. Introduction

Common variable immunodeficiency (CVID) is a frequent primary immune deficiency (PID) which consists of a heterogeneous group of disorders. It is more frequently seen in adults and characterized by impaired B cell differentiation resulting in hypogammaglobulinemia, normal or low numbers of B cells, and poor antibody response [1]. As the “variable” term implies, its clinical manifestation is heterogenous and includes recurrent infections, chronic pulmonary and gastrointestinal diseases, and chronic diarrhea as well as autoimmunity and increased susceptibility to malignancy [2].

Secondary amyloidosis, mostly reported in middle-aged men, is an uncommon complication of CVID [2]. Chronic and recurrent infections in patients with CVID may lead to extracellular deposition of serum amyloid A (SAA) protein fibrils [3]. Infectious diseases, bronchiectasis, cor pulmonale, respiratory distress, or tuberculosis, are the predisposing conditions for the development of amyloidosis in patients with CVID [4, 5]. Delay in the diagnosis of CVID or initiation of immunoglobulin replacement therapy or administration of insufficient doses of IVIg may contribute to the development of amyloidosis secondary to poor infection control [5].

In this paper, we describe an unusual case of a man with CVID who developed renal amyloidosis during his follow-up under IVIg replacement therapy.

## 2. Case Report

**Figure 1:**
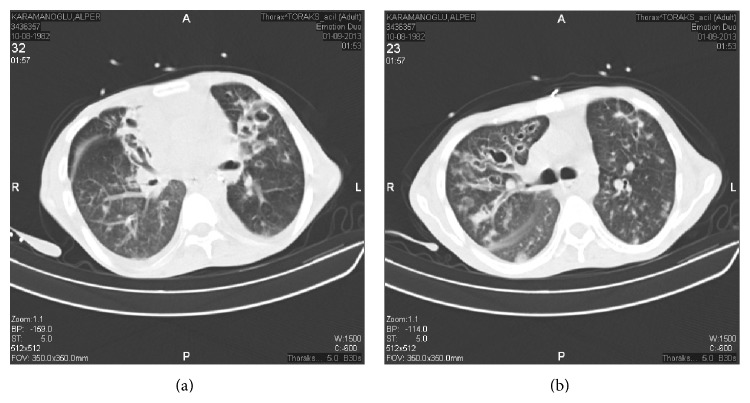
Thoracal CT of the patient shows bilateral bronchiectatic segments.

A 27-year-old male patient was referred to the division of Pediatric Immunology at Hacettepe University for further evaluation of recurrent sinopulmonary infections, chronic diarrhea, and hypogammaglobulinemia. He had been followed up with the diagnosis of bronchiectasis (Figure 1) since the age of 7 years and undergone two separate pulmonary lobectomy surgeries at the ages of 15 and 18 years. On presentation, his chief complaints were diarrhea for 9 months and loss of weight (~10 Kg) within the past 6 months. The microbiological evaluation of stool was negative for a bacteria, parasite, or* Cryptosporidium*. He had been evaluated by a colonoscopy at outside hospital and this was reportedly normal. His physical examination revealed normal vital signs, body mass index of 14 (weight: 43 Kg, height: 175 cm), clubbing in both hands and feet, right sided rales on lung auscultation, perforated nasal septum and left tympanic membrane, and diffuse erythematous, squamous plaques on the trunk, hands, and behind the ears, compatible with psoriasis. The family history revealed consanguinity.

**Figure 2:**
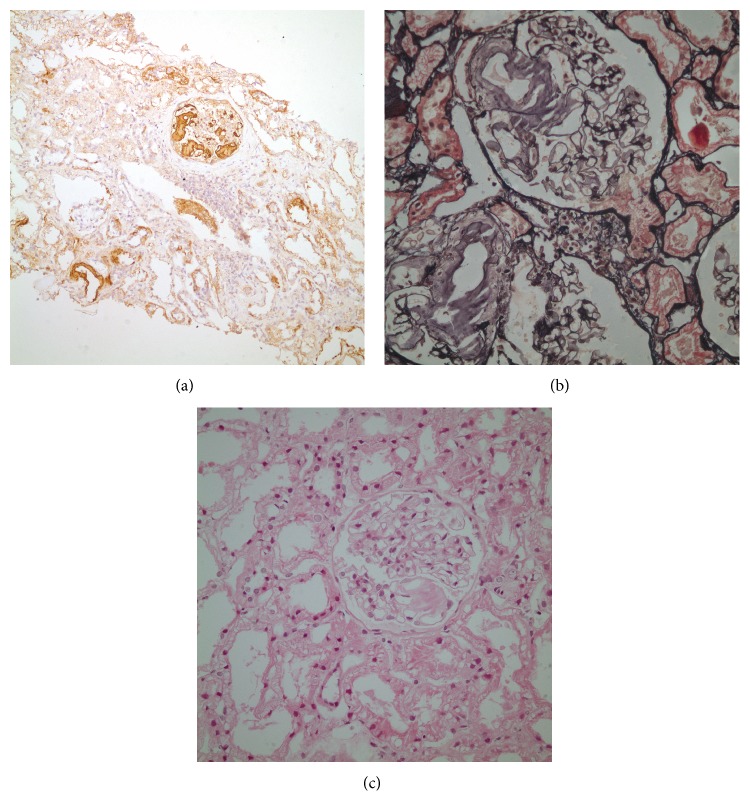
Immunohistochemical reactivity of deposited material with anti-amyloid A protein, note staining of glomeruli and vascular walls (a). Negative staining of deposited material (consistent with amyloid) silver stain (b). Focal mesangial widening due to deposition of amorphous acellular eosinophilic material consistent with amyloid. Note deposition of amyloid along the hilar arterioles (c).

Laboratory tests on admission showed hypogammaglobulinemia (IgG, 290 mg/dL [*n*: 913–1884]; IgA, 75 mg/dL [*n*: 139–378]; IgM, 314 mg/dL [*n*: 88–322]; total IgE, 1.93 mg/dL), anemia, and elevated erythrocyte sedimentation rate (65 mm/hr [*n*: 0–20]), and CRP level (16.25 mg/dL [*n*: 0–0.8]). There was no lymphopenia (ALC: 2600) or neutropenia (ANC: 8500) in the complete blood count. Total protein and serum albumin were normal (6.5 and 4.3 g/dL, resp.). Urine analysis was negative for proteinuria. Flow cytometry of peripheral blood revealed CD3 of 90%, CD4 of 19%, CD8 of 60%, CD16 + 56 of 7%, CD19 of 0%, and CD20 of 0%. In order to rule out X-linked agammaglobulinemia, Bruton tyrosine kinase (BTK) mutation was tested and found to be negative. Pneumococcal antibody response was absent. The clinical findings and laboratory workup did not let us to classify as CVID or combined immunodeficiency (the molecular analysis did not result yet). He was evaluated under the CVID umbrella and was treated with intravenous immunoglobulin therapy (IVIg) with the dose of 400 mg/kg once every 21 days for hypogammaglobulinemia and daily trimethoprim-sulfamethoxazole prophylactically for CD4 + T cell lymphopenia (absolute count: 392/mm^3^). The patient initially responded to the treatment well with cessation of pneumonia episodes and requirement for hospitalizations. He gained 10 Kg during the first year of follow-up (from 43 to 53 Kg). Two years later, at the age of 29 years, his IgG levels started declining progressively to levels around 200–300 mg/dL despite regular IVIg infusions. His serum albumin level decreased to 3.2 g/dL (*n*: 3.4–4.8) and he was found to have massive proteinuria with 1726.4 mg/day protein loss on a 24-hour urine collection. Urinary ultrasonography demonstrated increased echogenicity of renal parenchyma (grade 1). Rectal and gingival biopsies were performed with the suspicion of amyloidosis; however, this was negative. A renal biopsy was performed and pathology analysis revealed focal segmental accumulation of AA type amyloidosis in glomeruli and focal accumulation in the interstitium and vessel walls accompanied by tubular atrophy and increased mononuclear cells in the interstitium (Figure 2). Serum amyloid A (SAA) protein level was 330 mg/L (*n*: 0–10). Familial mediterranean fever was excluded with the absence of MEFV mutation. The patient was started on angiotensin receptor blocker and colchicine, since some previous reports showed decrease in proteinuria with colchicine treatment in patients with isolated renal amyloidosis [6, 7]. Three months/years later, the patient presented to our hospital with pneumonia leading to acute hypoxemic respiratory failure. He was treated with broad spectrum IV antibiotics, intubated and mechanically ventilated. Because of severe protein loss secondary to amyloidosis, his IVIg replacement dose was increased to 200 mg/kg twice monthly. His serum albumin levels progressively decreased to 1.3 g/dL despite all the aggressive measures and albumin infusions. The patient expired from sepsis and ARDS at the age of 33 years.

## 3. Discussion

SAA are acute phase proteins in the form of apolipoproteins associated with specific high-density lipoprotein (HDL), and they are expressed extrahepatically in the absence of HDL. Several cytokines (mainly IL-1, IL-6, and TNF), lipopolysaccharides, and transcription factors can induce SAA deposition [8]. During acute phase response, SAA increases the affinity of HDL for macrophages and adipocytes, binds to the extracellular matrix, shows chemoattractant activity for monocytes and lymphocytes, and stimulates the release of proinflammatory cytokines [9].

AA amyloidosis is well known to be a complication of chronic or recurrent inflammatory states seen with rheumatoid arthritis, inflammatory bowel disease, chronic infections, or periodic fever syndromes [9, 10]. The clinical important major sites for amyloid deposition are the kidneys, heart, gut, and liver. Renal involvement of amyloidosis may present with a range from symptomatic proteinuria to clinically apparent nephrotic syndrome [9]. If a patient's history and clinical manifestations raise suspicion for amyloidosis, a tissue biopsy should be performed in order to confirm the diagnosis. In case of a single organ involvement, tissue biopsy should be taken from the involved site. A fat pad aspiration biopsy is suggested as the initial biopsy technique for patients with more extensive involvement [11]. In our patient, renal biopsy was positive despite rectal and gingival biopsies negative for SAA deposition because the kidneys were the primarily affected organs. Increased SAA production and deposition in patients with CVID are most likely triggered by a defect in the control of inflammation in patients with CVID. Renal and/or intestinal loss of immunoglobulins due to involvement of these organs with amyloidosis leads to further worsening of hypogammaglobulinemia and subsequently increased frequency of infections despite IVIg replacement [12]. At the late course of the disease, our patient suffered from recurrent pulmonary infections and died from ARDS which can raise the suspicion for pulmonary amyloidosis which was reported in the literature before [13]. However, no tissue biopsy was performed to rule in or exclude this diagnosis in our patient.

Our patient had had several clinical features consistent with CVID since the age of 7 years. However, he was officially diagnosed with this disease at the age of 27 years. We believe that the years-long chronic and recurrent infections due to prolonged delay (~20 years) in the diagnosis and appropriate treatment of CVID led to renal amyloidosis and unfortunately subsequent mortality in our patient. Although the time course is still not well known, it is estimated that development of clinical amyloidosis may last for 8 to 14 years which is shorter than the untreated course in our patient [14]. Early diagnosis of primary immunodeficiency has prime importance in patients with recurrent symptoms. Ig replacement therapy is the mainstay of the management of PIDs and it should be started at appropriate doses and intervals as soon as possible after the diagnosis in order to prevent chronic inflammation and its complications. Patients should be kept under regular control by monitoring serum IgG levels and replacement with adequate Ig doses and prophylactic antibiotherapy to prevent infections. The possibility of amyloidosis should be suspected when the IgG levels cannot be maintained above 500 mg/dL.

Although Ig replacement therapy is generally started with the doses of 400 to 600 mg/kg every 21 or 30 days, for patients with bronchiectasis or chronic sinusitis, the dose can be as high as 600 to 800 mg/kg every 21 days as recommended in the literature [13]. Intravenous (iv) and subcutaneous (sc) routes of Ig replacement therapies have different pharmacokinetic profiles, and sc route may be preferred in patients with CVID, since IgG is first locally distributed, followed by slow diffusion into extravascular space from vascular space [14].

Delay in the diagnosis of CVID and initiation of IVIg replacement therapy in patients with recurrent infections can increase the risk of chronic inflammation resulting in an uncommon but life-threatening complication, amyloidosis. We believe that regular clinical follow-ups, control of infections, and adequate replacement of Ig may seem to prevent development of amyloidosis in patients with CVID. However, further research is needed to shed more light on the epidemiology, pathogenesis, screening, and management of amyloidosis in patients with CVID.
